# Serum matrix metalloproteinase-9 is a valuable biomarker for identification of abdominal and thoracic aortic aneurysm: a case-control study

**DOI:** 10.1186/s12872-018-0931-0

**Published:** 2018-10-29

**Authors:** Tan Li, Bo Jiang, Xuan Li, Hai-yang Sun, Xin-tong Li, Jing-jing Jing, Jun Yang

**Affiliations:** 1 Department of Cardiovascular Ultrasound, the First Hospital of China Medical University, No.155 Nanjing Bei Street, Heping District, Shenyang, 110001 China; 2 Department of Vascular and Thyroid Surgery, the First Hospital of China Medical University, Shenyang, 110001 China; 3 Tumor Etiology and Screening Department of Cancer Institute and General Surgery, the First Hospital of China Medical University, Shenyang, 110001 China

**Keywords:** Matrix metalloproteinase-9, Abdominal aortic aneurysm, Thoracic aortic aneurysm, C- reactive protein

## Abstract

**Background:**

Matrix metalloproteinase-9 (MMP9) has been reported to play a key role in the pathogenesis of aortic aneurysm. However, few studies have assessed serum MMP9 levels in both abdominal aortic aneurysm (AAA) and thoracic aortic aneurysm (TAA). In this study, we investigated the serum levels of MMP9 in aortic aneurysm to evaluate its predictive and diagnostic efficacy for AAA and TAA, and explored the association of MMP9 with circulating laboratory markers.

**Methods:**

A total of 296 subjects were enrolled, including 105 AAA patients, 79 TAA patients and 112 healthy controls. The levels of serum MMP9 were detected by enzyme-linked immunosorbent assay (ELISA).

**Results:**

Compared to control group, both AAA and TAA patients had higher serum MMP9 levels in the overall comparison and subgroup analysis based on subjects aged<65 years, either male or female, hypertension, non-diabetes and non-hyperlipidemia (all *P*<0.05). Moreover, MMP9 levels were significantly higher in TAA group than those in AAA group in the total comparison, and this discrepancy was also found in the non-diabetes, non-hyperlipidemia and aortic diameter ≥ 5.5 cm subgroup analysis. Serum MMP9 levels were influenced by age and hypertension. There was a positive association of serum MMP9 with CRP (*r* = 0.33, *P* < 0.001) and Hcy (*r* = 0.199, *P* = 0.033). Multiple logistic analyses showed that serum MMP9 was an independent risk factor for AAA and TAA. Based on receiver operating characteristic (ROC) analysis, the area under the curve (AUC) of MMP9 for predicting TAA was 0.83 with 70% sensitivity and 91% specificity, while the AUC of MMP9 to detect AAA was 0.69 and the sensitivity and specificity were 50% and 88%.

**Conclusions:**

Serum MMP9 was closely related to the existence of aortic aneurysms and could be a valuable marker for the discrimination of aortic aneurysm, especially for TAA.

## Background

Aortic aneurysm is a complex and dangerous vascular disease that results from the multifactorial interaction of genetic and environmental factors [1]. According to anatomical locations, aortic aneurysm is generally divided into abdominal aortic aneurysm (AAA) and thoracic aortic aneurysm (TAA). They may share some similarities in pathogenesis and histological phenotypes that both involve the metabolic imbalance and progressive weakening of aortic wall [2, 3], but not for syndrome and bicuspid valve associated TAA. Matrix metalloproteinase-9 (MMP9) is a gelatinase with proteolytic activity on extracellular matrix degradation in aortic wall and its excessive production can lead to progressive aortic remodeling and dilatation [2]. Any cause increasing the activity of aortic endothelial cells, smooth muscle cells and infiltrating inflammatory cells can produce a large amount of MMP9 released into blood circulation [4].

Evidence has demonstrated higher circulating MMP9 levels in AAA patients [5, 6]. As for TAA, studies often focused on the gene variation and tissue expression of MMP9 [7, 8], but much less is known about the association between serum MMP9 and TAA. A study by Meffert et al. suggested no different expression of serum MMP9 between TAA with hyperlipidemia and without hyperlipidemia [9]. Based on small sample sizes, Tsarouhas et al. revealed that there were increased MMP9 levels in TAA serum and tissue [10], however, Karapanagiotidis et al. showed that TAA patients had lower serum MMP9 levels [11]. To our knowledge, there were no studies available on detailed comparison of serum MMP9 levels in AAA and TAA, and the performance of serum MMP9 for identification of aortic aneurysm is still unknown. To date, circulating biomarkers, such as C-reactive protein (CRP), homocysteine (Hcy) and Cystatin C (Cys-c), have been analyzed in aortic aneurysmal diseases, but it remains unclear whether the studied biomarkers are correlated with serum MMP9.

In the present study, we attempted to explore the overall and stratified comparative differences in serum MMP9 levels and evaluate its potential clinical applicability for predicting AAA and TAA. Meanwhile, we intended to determine the relationship between serum MMP9 and other laboratory markers to discuss their possible interaction relevant for aortic aneurysm.

## Methods

### Subjects

This was a single center, case-control study. A total of 105 AAA patients, 79 TAA patients and 112 controls were recruited from the First Hospital of China Medical University between October 2016 and October 2017. Thoracoabdominal aortic aneurysms and AAA cases with extension to the iliac artery were not included in this study. The diagnosis of all patients was based on the computed tomography angiography (CTA). Exclusion criteria included the subjects accompanied by congenital genetic disorders, severe cardiovascular diseases, autoimmune diseases, severe organ failure, infectious diseases, malignant tumors, hematological system diseases, previous aortic surgery or received non-steroidal anti-inflammatory drugs or steroids. Demographic data, risk factors and laboratory parameters were obtained from clinical records. Hypertension was defined as having a systolic blood pressure (SBP) ≥ 140 mmHg and/or having a diastolic blood pressure (DBP) ≥90 mmHg and/or being under antihypertensive treatment. Diabetes was defined as fasting serum glucose (FPG) ≥7 mmol/L (126 mg/dL) and (or) being on treatment for diabetes [12]. Hyperlipidemia was defined as serum total cholesterol (TC) ≥6.22 mmol/L (240 mg/dL), or serum triglyceride (TG) ≥2.26 mmol/L (200 mg/dL), or serum low-density lipoprotein cholesterol (LDL-C) ≥4.14 mmol/L (160 mg/dL) [13]. For subjects with AAA or TAA, the maximal aortic diameter was assessed via CTA using the average of three measurements. This study was approved by the Ethics Committee of the First Hospital of China Medical University (Shenyang, China). Written informed consent was obtained from each subject.

### Detection of serum MMP9

Blood samples were collected using standardized sterile tubes and centrifuged at 3500 r/min for 10 min at 4 °C, and the serum was separated, and stored at − 80 °C until being assayed. Serum MMP9 levels were measured by enzyme-linked immunosorbent assay (ELISA) using MMP9 ELISA kits (Wuhan Boster Biotechnology Company, Wuhan, Hubei, China), according to the manufacturer’s protocol.

### Statistical analysis

All statistical analyses were performed using SPSS version 17.0 software. Continuous variables were reported as mean values and standard deviations, and categorical variables were represented as numbers and percentages. Differences among categories were evaluated using ANOVA, independent-sample t-test or χ^2^ test as appropriate. Spearman’s rank correlation test was used to examine the associations of serum MMP9 levels with laboratory markers and maximal aortic diameter. Multiple logistic regression models were performed to determine the predictive value of serum MMP9 in AAA or TAA risk with the adjustment for the potential confounding factors. Receiver operating characteristic (ROC) curves and the area under the curve (AUC) were used to evaluate the diagnostic effects of serum MMP9 and to determine appropriate cut-off points. A two-sided *P*<0.05 was considered statistically significant.

## Results

### Characteristics of the study subjects

The detailed clinical characteristics of the cases and controls were described and compared in Table 1. Male members made up a larger proportion in three groups. AAA and TAA patients tended to have higher heart rate, leucocyte count and blood pressure compared with control subjects.

**Table 1:** Clinical characteristics of the study subjects

Variables	Control	AAA	TAA
	*N* = 112	*N* = 105	*N* = 79
Age, years	62.32 ± 11.23	66.75 ± 10.28^#^	59.04 ± 11.55^*†^
Males, n (%)	83(74.1%)	78(74.2%)	57(72.2%)
Height, cm	167.70 ± 7.20	169.49 ± 7.06	169.61 ± 7.10
Weight, kg	70.12 ± 10.13	66.40 ± 12.36^#^	73.38 ± 11.80^†^
Heart rate, bmp	73.58 ± 9.67	79.70 ± 13.58^*#*^	79.92 ± 15.65^*^
Leucocyte, ×10^9^/L	5.82 ± 1.77	7.94 ± 3.53^#^	11.16 ± 4.87^*†^
Thrombocyte, ×10^9^/L	221.51 ± 56.72	218.65 ± 81.38	207.88 ± 64.66
SBP, mmHg	134.48 ± 17.19	141.79 ± 19.56^#^	152.69 ± 28.98^*†^
DBP, mmHg	77.70 ± 11.67	80.65 ± 12.22	86.44 ± 18.33^*^
TC, mmol/L	4.90 ± 0.87	4.75 ± 1.01	4.41 ± 0.91^*†^
TG, mmol/L	1.53 ± 0.88	1.40 ± 1.06	1.35 ± 0.90
LDL-C, mmol/L	3.13 ± 0.77	3.15 ± 0.91	2.68 ± 0.74^*†^
HDL-C, mmol/L	1.32 ± 0.34	1.04 ± 0.31^#^	1.13 ± 0.37^*^
FPG, mmol/L	5.55 ± 1.63	5.74 ± 1.56	6.43 ± 1.84^*†^
CRP, mg/L	–	32.97 ± 51.71	64.95 ± 47.47^†^
Hcy, umol/L	–	18.60 ± 11.60	18.52 ± 12.77
Cys-c, mg/L	–	1.14 ± 0.44	1.40 ± 0.76^†^
Max. aortic diameter, cm	–	5.64 ± 1.61	5.28 ± 0.95

*AAA* abdominal aortic aneurysm, *TAA* thoracic aortic aneurysm

*P*^#^: AAA vs. Control, P^*^: TAA vs. Control, *P*^†^: TAA vs. AAA

### Serum MMP9 levels between different groups in the total and stratified comparisons

Serum MMP9 levels in control, AAA and TAA groups were 258.79 ± 133.00, 333.45 ± 138.57 and 385.17 ± 109.23 ng/ml, respectively. In the total comparison, there were significantly higher serum MMP9 levels in AAA and TAA groups than those in control group, while TAA subjects had higher serum MMP9 levels when compared with AAA patients(all *P*<0.05).

Furthermore, we compared serum MMP9 levels between different groups stratified by age, gender, hypertension, diabetes or hyperlipidemia status and maximal aortic diameter, as shown in Table 2. The results showed that MMP9 levels were significantly increased from control to AAA to TAA group in the subjects aged<65 years. Compared to control group, AAA and TAA patients had increased MMP9 levels in either male or female, and this discrepancy was also found in hypertension status. Meanwhile, serum MMP9 levels in TAA patients were significantly higher than those in AAA subjects in male subgroup comparison. However, in the non-diabetes and non-hyperlipidemia status, MMP9 levels were obviously higher from control to AAA to TAA group (all *P *< 0.05). When stratified by maximal aortic diameter, TAA patients tended to have higher MMP9 levels than AAA cases only in the subgroup with max. Aortic diameter ≥ 5.5 cm.

**Table 2:** Serum MMP9 levels in age, gender, risk factors and maximal aortic diameter

		Control			AAA			TAA		*P* ^*#*^	*P* ^*^	*P* ^*†*^
		MMP9(ng/ml)	*P*		MMP9(ng/ml)	*P*		MMP9(ng/ml)	*P*			
Total		258.79 ± 133.00			333.45 ± 138.57			385.17 ± 109.23		<0.001	<0.001	0.015
Age	<65y(60)	200.78 ± 139.20	<0.001	<65y(47)	299.44 ± 160.84	0.029	<65y(52)	378.63 ± 96.80	0.464	0.004	<0.001	0.013
	≥65y(52)	325.73 ± 86.94		≥65y(58)	361.01 ± 111.50		≥65y(27)	397.77 ± 131.00		0.087	0.005	0.143
Gender	male(83)	260.57 ± 135.13	0.812	male(78)	328.11 ± 144.39	0.505	male(55)	390.09 ± 110.19	0.548	0.008	<0.001	0.017
	female(29)	253.69 ± 128.88		female(27)	348.88 ± 121.38		female(24)	373.90 ± 108.47		0.004	0.001	0.462
Hypertension	Yes(46)	291.13 ± 114.98	0.026	Yes(64)	362.45 ± 140.89	0.007	Yes(53)	405.44 ± 91.35	0.038	0.013	<0.001	0.141
	No(66)	236.25 ± 140.71		No(41)	288.17 ± 123.34		No(26)	343.87 ± 131.23		0.053	0.001	0.099
Diabetes	Yes(14)	308.00 ± 110.55	0.11	Yes(23)	316.37 ± 153.51	0.506	Yes(33)	379.07 ± 124.77	0.677	0.997	0.177	0.301
	No(98)	251.76 ± 134.93		No(82)	338.24 ± 134.72		No(46)	389.55 ± 97.79		<0.001	<0.001	0.043
Hyperlipidemia	Yes(26)	273.10 ± 128.94	0.494	Yes(23)	359.34 ± 151.80	0.313	Yes(16)	402.51 ± 100.01	0.481	0.099	0.002	0.644
	No(86)	253.56 ± 134.85		No(82)	326.19 ± 134.73		No(63)	380.77 ± 111.77		0.002	<0.001	0.026
Max. aortic diameter	NA	–	–	<5.5(60)	334.62 ± 131.26	0.923	<5.5(55)	371.19 ± 112.91	0.085	–	–	0.114
	NA	–		≥5.5(45)	331.89 ± 149.26		≥5.5(24)	417.22 ± 94.82		–	–	0.005

*AAA* abdominal aortic aneurysm, *TAA* thoracic aortic aneurysm

*P*^#^: AAA vs. Control, P^*^: TAA vs. Control, *P*^†^: TAA vs. AAA

### Influence of age, gender, risk factors and maximal aortic diameter on serum MMP9

Table 2 also showed the comparison results in the individual group. We found that the≥65 years group had much higher serum MMP9 levels compared with the <65 years group in the control and AAA group. Serum MMP9 levels were significantly higher in subjects with hypertension than those without hypertension in each group (all *P*<0.05). However, there were no significant differences in the serum MMP9 levels between male and female, diabetes and non-diabetes, hyperlipidemia and non- hyperlipidemia, max. Aortic diameter ≥ 5.5 cm and <5.5 cm groups.

### Correlation of serum MMP9 with laboratory biomarkers and maximal size of aneurysm

**Fig. 1:**
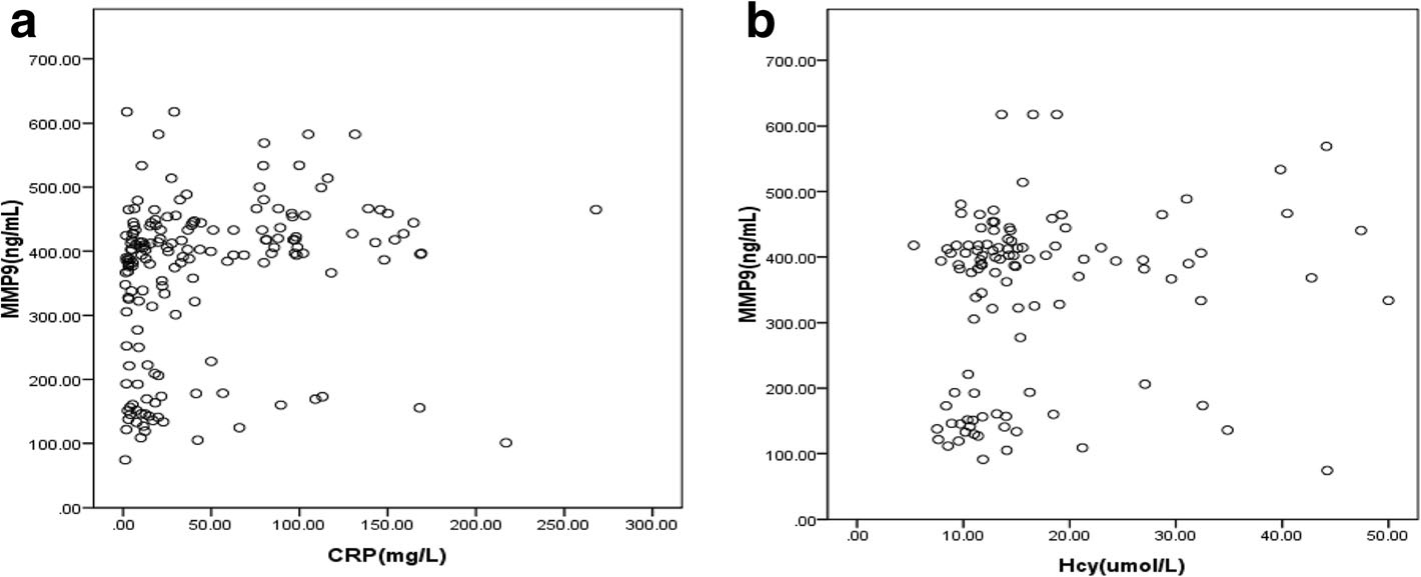
Scatter plots for the association of serum MMP9 with CRP(**a**) and Hcy(**b**)

We evaluated a possible correlation between MMP9 levels and CRP, Cys-c, Hcy and maximal aortic diameter. Serum MMP9 levels had a positive association with the concentration of CRP(*r* = 0.330, *P* < 0.001) and Hcy(*r* = 0.199, *P* = 0.033) (Fig. 1). However, there was no significant relationship of MMP9 levels with Cys-c(*r* = 0.097, *P* = 0.272) and maximal aortic diameter(*r* = 0.008, *P* = 0.918).

### Predictive and diagnostic value of serum MMP9 for aortic aneurysm

We further performed multiple logistic regressions to evaluate the risk prediction value of serum MMP9 for AAA and TAA under different adjustment models, as shown in Table 3. When all potential confounding factors were adjusted, serum MMP9 was still significantly associated with AAA risk (OR = 1.004 per unit increase, 95% CI = 1.001–1.007, *P* = 0.018) and TAA risk (OR = 1.014 per unit increase, 95% CI = 1.006–1.022, *P *< 0.001).

**Table 3:** Multiple logistic regression analysis of serum MMP9 levels for AAA and TAA risk

Variables	AAA		TAA	
	OR(95%CI)	*P*	OR(95%CI)	*P*
Model 1				
MMP9, ng/mL	1.004(1.002–1.006)	<0.001	1.010(1.007–1.013)	<0.001
Model 2				
MMP9, ng/mL	1.004(1.001–1.007)	0.006	1.012(1.007–1.018)	<0.001
Model 3				
MMP9, ng/mL	1.004(1.001–1.007)	0.018	1.014(1.006–1.022)	<0.001

Model 1: age and gender were adjusted

Model 2: Model 1 plus height, weight, heart rate, leucocyte and thrombocyte

Model 3: Model 2 plus hypertension, diabetes and hyperlipidemia

**Fig. 2:**
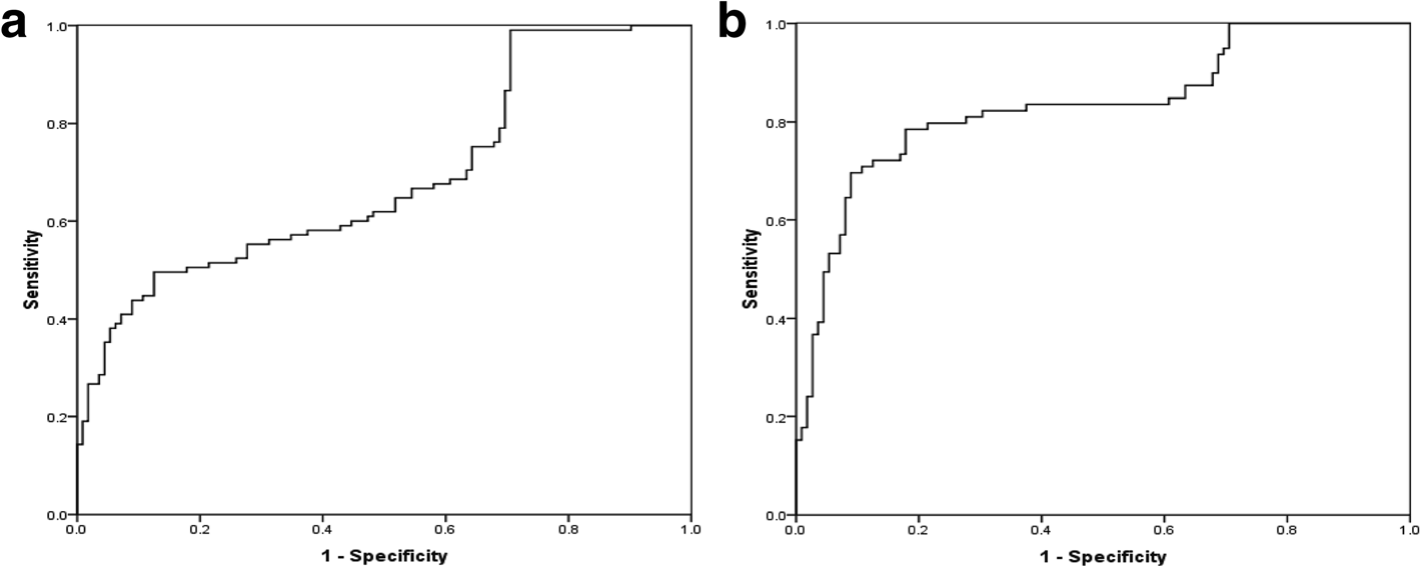
ROC curve for serum MMP9 levels to predict AAA (**a**) and TAA (**b**)

In addition, the ROC curves of MMP9 levels for predicting AAA and TAA (Fig. 2). The ROC curve analysis illustrated that MMP9 levels had strong diagnostic value for TAA with the AUC of 0.83(95% CI: 0.77–0.90; *P* < 0.001) and an optimal cut-off point of 393.00 ng/ml associated with corresponding validity parameters of 70% sensitivity and 91% specificity. However, the AUC of MMP9 for predicting AAA was 0.69(95% CI: 0.62–0.76; *P* < 0.001) and MMP9 ≥ 385.32 ng/ml had a sensitivity of 50% and a specificity of 88%.

## Discussion

Serum MMP9 levels represent the leakage of enzyme into the bloodstream during periods of matrix catabolism and its elevation may reflect a more active state of degeneration of the aortic wall. In the current study, our results suggested higher MMP9 levels in either AAA or TAA group than those in control group. Interestingly, we also found that TAA patients tended to have higher MMP9 levels than AAA subjects in the overall comparison, which might depend on their different embryological feature, wall mechanics and arterial hemodynamics [2]. Compared to the abdominal aorta, thoracic aorta has thicker aortic media [14, 15] and a higher degree of wall shear stress [16, 17], which were possibly linked to higher MMP9 production for aneurysm formation.

In the subgroup comparisons stratified by age, gender, hypertension, diabetes and hyperlipidemia, we found that both AAA and TAA patients had higher MMP9 levels in the subjects aged <65 years, either male or female, and hypertensive status compared with controls. In addition, MMP9 levels showed to increase from control to AAA to TAA group in the non-diabetes and non-hyperlipidemia status. Hypertension is a well-known risk factor associated with aortic aneurysm. However, although diabetes and hyperlipidemia are recognized cardiovascular risk factors strongly associated with most acquired cardiovascular pathologies, they seem to be relatively weak risk factors for aortic aneurysm. Some studies indicated that the presence of diabetes had a reduced risk for aortic aneurysm, resulting from decreased MMPs production and activation in the aortic wall [18, 19]. Recent research also found that diabetes inhibited experimental aortic aneurysm progression through reducing macrophage infiltration and medial elastolysis, and lowering serum glucose could diminish its protective effects [20, 21]. Hyperlipidemia and its associated effect on extracellular matrix have also been demonstrated to limit AAA formation in mice [21], and showed a negative association with AAA risk [22, 23]. These observations identified the protective role of diabetes and hyperlipidemia in aneurysm formation, which might partly account for our findings that the different MMP9 levels were obviously reflected in non-diabetic and non-hyperlipidemia subgroup analysis. Furthermore, we analyzed the effects of traditional cardiovascular risk factors on serum MMP9 levels. We found that subjects aged ≥65 years had higher MMP9 levels than those aged <65 years. Additionally, hypertensive participants exhibited much higher serum MMP9 levels than non-hypertensive ones in each group.

Maximal aortic diameter, as a surrogate clinical marker of the growth rate, has been used to discuss its potential correlation with MMP9 levels [16]. MMP9 activity varied with aortic diameter in AAA, and its expression was reported to be elevated in aneurysms with a diameter of 5.0 to 6.9 cm [24]. Freestone et al. [25] indicated an increased activity of MMP9 in aneurysms with a diameter ≥ 5.5 cm. On the contrary, neither Hovsepian nor Eugster had observed a significant correlation between serum MMP9 and aneurysm size of AAA [26, 27]. A recent study also suggested no relationship between serum MMP9 and TAA diameter [9]. In our study, serum MMP9 levels were not influenced by maximal aortic diameter in either AAA or TAA group, however, TAA patients were prone to have higher MMP9 levels than AAA subjects in aortic diameter ≥ 5.5 cm subgroup comparison.

Various systemic laboratory diagnostic biomarkers have been investigated and linked to the risk for aortic aneurysm or its outcomes, such as CRP, Hcy and Cys-c. CRP, a sensitive and non-specific inflammatory marker, has been recognized as an independent risk factor in the detection of vascular inflammation [4]. It has been considered to be associated with aortic aneurysm presence and progression [28, 29]. Moreover, CRP can induce MMP9 production in human mononuclear cells in a concentration-dependent manner [30, 31]. Hcy, a sulfur containing amino acid, has been suggested to play a key role in aortic aneurysm [32, 33]. Moreover, Hcy can produce marked vascular remodeling and elastolysis of the aortic wall by excessive MMPs production and activation [10, 34]. Above evidence might explain our findings that serum MMP9 levels were positively correlated with circulating CRP and Hcy, which could partly reflect the fact that multiple biomarkers responded to aortic aneurysm related events. Although circulating Cys-c has been proved to be related to AAA and may favor proteolysis in the pathogenesis of AAA [35], we found no association of serum MMP9 with Cys-c.

To further assess the clinical application value of serum MMP9 in aortic aneurysm, we explored its predictive and diagnostic efficacy for identifying AAA or TAA. In multiple logistic analyses after adjusting the possible confounders, we demonstrated that serum MMP9 was an independent risk factor for the existence of AAA or TAA. With a low expected incidence, the focus for a biomarker should primarily be on specificity. Based on ROC curves, elevated MMP9 levels suggested a higher specificity than sensitivity in recognizing either AAA or TAA, which represented that serum MMP9 conferred a crucial role in safely ruling out aortic aneurysm. Moreover, AUCs, cut-off points for serum MMP9, and the sensitivity and specificity were much better with its use for predicting TAA than AAA. Therefore, serum MMP9 may be a valuable diagnostic biomarker for aortic aneurysm, especially for TAA.

Our study has some limitations. First, our sample size was small, and a more precise localization and detailed classification for TAA cases were lacking. Second, the serial measurements of MMP9 levels for evaluating dynamic changes were not investigated. Third, there were no complete records of potential confounders relevant to aortic aneurysm and MMP9 levels, such as the history of smoking and drinking. In addition, histological samples and MMP9 tissue expression were not investigated in this study. Further prospective studies based on larger scale cohorts will be needed to validate the clinical applicability of serum MMP9 on the identification of aortic aneurysm initiation and progression in the future practice.

## Conclusions

Our results suggested that MMP9 levels were significantly increased in AAA and TAA group. Furthermore, we also found TAA patients tended to have higher levels of MMP9 than AAA subjects in the overall comparison, and in the non-diabetes, non-hyperlipidemia and aortic diameter ≥ 5.5 cm subgroup analysis. Moreover, MMP9 levels were affected by age and hypertension, and were positively associated with circulating CRP and Hcy. Multiple logistic analyses further suggested that serum MMP9 was an independent risk factor for aortic aneurysm. Serum MMP9 showed a high specificity for the diagnosis of either AAA or TAA. Therefore, MMP9 might be a useful biomarker with clinical predictive and diagnostic value for aortic aneurysm, especially for TAA.

## Abbreviations

AAA: Abdominal aortic aneurysm
AUC: Area under the curve
CRP: C-reactive protein
CTA: Computed tomography angiography
Cys-c: Cystatin C
DBP: Diastolic blood pressure
ELISA: Enzyme-linked immunosorbent assay
FPG: Fasting serum glucose
Hcy: Homocysteine
HDL-C: High-density lipoprotein cholesterol
LDL-C: Low-density lipoprotein cholesterol
MMP9: Matrix metalloproteinase-9
ROC: Receiver operating characteristic
SBP: Systolic blood pressure
TAA: Thoracic aortic aneurysm
TC: Total cholesterol
TG: Triglyceride
